# Causally-Informed Instance-Wise Feature Selection for Explaining Visual Classifiers

**DOI:** 10.3390/e27080814

**Published:** 2025-07-29

**Authors:** Li Tan

**Affiliations:** Adobe, San Francisco, CA 94103, USA; lit@adobe.com

**Keywords:** interpretability, causality, conditional mutual information, matrix-based Rényi’s α-order entropy functional

## Abstract

We propose a novel interpretability framework that integrates instance-wise feature selection with causal reasoning to explain decisions made by black-box image classifiers. Instead of relying on feature importance or mutual information, our method identifies input regions that exert the greatest causal influence on model predictions. Causal influence is formalized using a structural causal model and quantified via a conditional mutual information term. To optimize this objective efficiently, we employ continuous subset sampling and the matrix-based Rényi’s α-order entropy functional. The resulting explanations are compact, semantically meaningful, and causally grounded. Experiments across multiple vision datasets demonstrate that our method outperforms existing baselines in terms of predictive fidelity.

## 1. Introduction

As machine learning systems are increasingly deployed in high-stakes domains, the need for transparent and trustworthy model behavior becomes critical, particularly for black-box classifiers with opaque decision-making processes. In the field of computer vision, a common strategy for enhancing interpretability involves generating saliency maps that visually highlight the contribution of each pixel to the model’s prediction. While gradient-based techniques have gained popularity due to their intuitive appeal, they often suffer from instability, lack of robustness, and sensitivity to perturbations in both models and data [1,2]. These limitations undermine their reliability in critical applications. To address these shortcomings, recent research has introduced the paradigm of instance-wise feature selection, which shifts the focus from continuous attribution to the discrete selection of the most informative input features, such as individual pixels or image patches, on a per-instance basis [3,4,5]. This approach not only yields more compact and human-interpretable explanations but also lays the foundation for integrating causal reasoning into model interpretation by emphasizing features that actively influence the prediction, rather than merely correlating with it.

One of the earliest and most influential approaches in the domain of instance-wise feature selection is L2X [6], which formulates the problem as selecting a fixed number of features that maximize mutual information with respect to the model’s output. This method is based on the idea that the most informative features, in terms of their statistical dependence on the prediction, can offer valuable insight into the model’s decision process. However, L2X requires the number of selected features to be predefined, which can limit its adaptability across different datasets or applications where the optimal number of relevant features varies. To address this constraint, INVASE [7] was subsequently introduced as an extension that eliminates the need to specify the subset size in advance. It employs a reinforcement learning-based strategy to dynamically determine the number and identity of important features for each input instance. Despite this architectural enhancement, both L2X and INVASE share a common limitation: they rely on mutual information as their primary objective for optimization.

While mutual information is effective in measuring statistical dependency, it does not necessarily reflect causal relationships. In particular, a feature may exhibit high mutual information with the output variable simply because it is correlated with other influential inputs rather than exerting a direct causal influence on the model’s decision [8,9]. As a result, explanations generated by such methods may emphasize features that are associated with the output but are not causally responsible for the prediction. This can lead to misleading or incomplete interpretations, especially in domains where understanding causal mechanisms is essential for downstream decision-making, such as medicine, finance, or safety-critical systems [10,11].

In this work, we argue that a robust explanation method should prioritize the identification of features that exert a direct causal influence on a model’s prediction. Specifically, we seek to select those input components whose inclusion or removal leads to meaningful changes in the model’s output. Our core hypothesis is that the most causally influential features are typically sparse and class-specific, capturing only the essential parts of the input that truly drive the decision. In contrast to prior methods such as L2X and INVASE, which may conflate statistical association with causation, our approach integrates a principled, information-theoretic measure of causal influence [12,13] into the feature selection process. This yields explanations that are not only compact and interpretable but also more faithful to the model’s underlying decision-making behavior. To formalize this notion, we adopt a structured causal model (SCM) framework [8] and employ an information-theoretic metric [12,13] as a rigorous measure of causal strength.

Although this new measure provides a theoretically sound basis for quantifying causal effects, its direct application in deep learning models can be computationally intensive and difficult to optimize. To make the framework practical, we further introduce the recently proposed matrix-based Renyi’s α-order entropy functional [14] to estimate causal strength while enabling efficient training within modern machine learning pipelines. Based on this formulation, we construct an instance-wise feature selector that identifies causally important subsets of input features. To facilitate gradient-based optimization, we adopt a continuous subset sampling strategy using the Gumbel–Softmax technique [15]. This allows the selector network to learn which features are causally influential through standard backpropagation, without resorting to reinforcement learning or combinatorial search.

We validate the proposed approach through a series of experiments on three benchmark vision datasets: MNIST, Fashion-MNIST, and CIFAR-10. For each dataset, we train a black-box classifier and apply our explainer to generate instance-specific explanations. To assess the effectiveness of the selected features, we conduct both qualitative analyses and quantitative evaluations. In our comparisons, we include several popular explanation methods, namely L2X, Grad-CAM, and Saliency maps, which represent diverse families of interpretability techniques. For quantitative comparison, we employ the post-hoc accuracy [5], which measures the predictive consistency of the model when restricted to the selected features. Our experimental findings consistently demonstrate that the proposed method produces more causally meaningful explanations compared to existing approaches. In particular, our method maintains competitive post-hoc accuracy, indicating that the causal features also preserve the classifier’s decision performance. These results highlight the advantages of combining instance-wise selection with causal analysis, and illustrate the potential of our approach for improving the transparency and reliability of black-box decision-making systems.

To summarize, our main contributions are as follows:

- We propose a novel instance-wise feature selection framework that is explicitly grounded in causal inference. By adopting the definition of causality based on the “do”-operator or intervention from [12] and establishing its connection to conventional conditional mutual information in probability theory, our method prioritizes features that have a genuine causal impact on the model’s prediction.
- We develop a practical training strategy by approximating the causal objective using conditional mutual information and enabling reliable estimation with the matrix-based Rényi’s α-order entropy functional and differentiable subset selection through the Gumbel-Softmax technique.
- We evaluate our approach on three standard vision datasets. Our method demonstrates improved performance compared to several popular interpretability baselines, especially in capturing causal influence.

## 2. Related Work

Model interpretability is a critical aspect of trustworthy machine learning, particularly in high-stakes domains such as healthcare, finance, and scientific discovery. A wide array of post-hoc explainability techniques have been developed to shed light on the decision-making process of complex models. These approaches can be broadly categorized into gradient-based methods, Shapley-value-based explanations, instance-wise feature selection, and information bottleneck-based frameworks.

Gradient-based methods aim to identify influential input features by analyzing the gradients of the model’s output with respect to its input. Classic approaches include Saliency Maps [16], which visualize the absolute value of gradients, and Grad-CAM [17], which backpropagates importance scores from intermediate feature maps in convolutional neural networks. While these methods are computationally efficient and provide visual intuitions, they are often criticized for their instability and sensitivity to input perturbations [1]. Furthermore, gradient-based attributions do not offer guarantees of causality, and they may reflect merely local sensitivity rather than true explanatory factors.

Shapley-value-based approaches derive from cooperative game theory, where the goal is to assign each feature an importance value based on its marginal contribution to the prediction. SHAP [18] is one of the most popular techniques in this category, offering a unified framework that satisfies desirable axioms such as local accuracy, missingness, and consistency. Despite its theoretical appeal, SHAP typically relies on strong independence assumptions or background distribution sampling, which can misrepresent causal relationships between features. Additionally, the computational cost of exact Shapley value computation grows exponentially with the number of input features, often requiring sampling-based approximations.

Instance-wise feature selection methods seek to identify a minimal subset of features that are sufficient for the model to make accurate predictions on a per-sample basis. Notable examples include L2X [6], which uses mutual information to guide feature selection through a variational approximation, and INVASE [7], which frames feature selection as a reinforcement learning problem. These methods offer fine-grained interpretability by adapting explanations to individual data points. However, their primary objective is predictive sufficiency rather than uncovering causal mechanisms, and they may capture spurious correlations present in the training data.

In recent years, the Information Bottleneck (IB) principle [19] has emerged as a popular method for instance-wise feature selection in both post-hoc and intrinsically interpretable visual classifiers [3,4]. The general objective is typically formulated as:

(1) $$\min I\left(X;X_{S}\right)-\beta I\left(Y;X_{S}\right),$$

where $X_{S}$ is a subset of features from *X*, and *Y* represents either the ground-truth label [4] or the prediction of the classifier being explained [3,20]. This idea has recently been extended to the Causal Information Bottleneck (CIB) framework [21], which aims to ensure that the selected features are causally related to the prediction. The authors of [21] also introduced causal information gain [22] to quantify causal strength. However, these methods are typically validated only on synthetic data or simple vectorized features.

Our objective, as will be presented in Section 3.3, differs from the standard IB formulation described above. Moreover, we show that causal strength can be effectively measured using a simple conditional mutual information term, rather than relying on causal information gain, which requires the use of the do-operator.

In summary, while existing interpretability methods have made significant strides, they either lack robustness, causal guarantees, or the ability to produce personalized explanations. Our work aims to bridge this gap by introducing an instance-wise explanation framework that explicitly incorporates causal reasoning, providing both accurate and causally meaningful interpretations.

## 3. Methodology

### 3.1. Problem Setup

**Figure 1:**
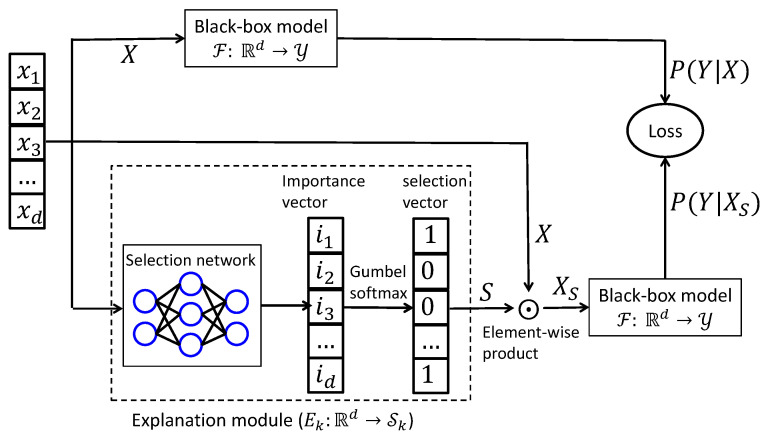
Diagram of instance-wise causal feature selection. To generate explanations, each training sample is processed by an explainer network that produces a sparse binary mask of length *d*, indicating the top-*k* features selected for interpretation. This is achieved by first computing a causal influence score using the matrix-based Rényi’s α-order entropy functional, followed by differentiable subset sampling using the Gumbel–Softmax trick to enable gradient-based optimization. The original input *X* and its masked counterpart $X_{s}$ are then passed through the black-box model to estimate their respective conditional outputs. These outputs are used to evaluate the objective function, and gradients are propagated back to update the parameters of the explainer network.

We focus on interpreting a pretrained, non-transparent classifier $F:R^{d}\to Y$ by identifying, for each input $x\in R^{d}$, a subset of *k* features whose presence most significantly influences the model’s output. To this end, we train an explainer network $E:R^{d}\to S_{k}$, where $S_{k}$ is the set of binary masks with *k* ones. The true explanation is obtained by inverting this mask, thus highlighting the *k* most relevant features (see Figure 1).

We adopt a structural causal modeling perspective, where the black-box model is abstracted as a Directed Acyclic Graph (DAG) with unidirectional links from input nodes to output variables. This view supports the interpretation of feature-to-output influence using a formal notion of causality.

To quantify the causal impact of a subset of features S⊂{1,…,d}, we adopt an information-theoretic measure of causal influence (detailed in the next subsection), which ultimately reduces to the conditional mutual information $I(X_{S};Y|X_{\bar{S}})$, enabling tractable optimization.

### 3.2. Quantifying Causal Strength

**Definition** **1** ([12]). *Let U, V, and W be disjoint subsets of nodes. The information flow from U to V imposing W, denoted I(U→V∣W), is*

(2) $$E_{w∼W}\left[\int_{U}p\left(u|do\left(w\right)\right)\int_{V}p\left(v|do\left(u\right),do\left(w\right)\right)\log\frac{p(v|do(u),do(w))}{\int_{u^{\prime }}p\left(u^{\prime }|do\left(w\right)\right)p\left(v|do\left(u^{\prime }\right),do\left(w\right)\right)}\;dV\;dU\right],$$

*where do(w) represents an intervention in a model that fixes w to a specified value, regardless of the values of its parents [8].*

Despite the elegant and intuitive formulation of causality in Equation (2), it remains challenging to estimate in practice, especially in the context of deep learning. To address this, we first establish its connection to conventional conditional mutual information, as shown in Proposition 1.

**Figure 2:**
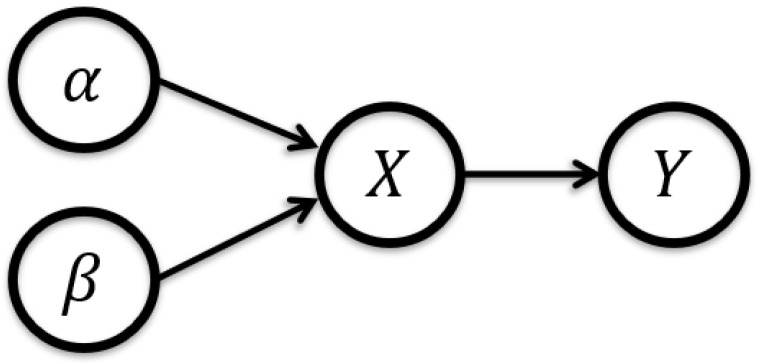
Directed Acyclic Graph (DAG) describing our causal model. In our case, α refers to the causal feature subset $X_{S}$, whereas β refers to the complement subset $X_{\bar{S}}$.

**Proposition** **1.** *Information flow in our DAG. The information flow from α to Y imposing β in the DAG of Figure 2 coincides with the mutual information of α and Y conditioned on β,*

(3) I(α→Y∣do(β))=I(α;Y∣β),

*where conditional mutual information is defined as $I\left(X;Y|Z\right)=E_{X,Y,Z}\left[\log\frac{p(x,y|z)}{p(x|z)p(y|z)}\right]$.*

**Proof.**  The proof follows from the “action/observation exchange” rule of the do-calculus [8] (Thm. 3.4.1). This rule asserts that p(y∣do(x),do(z),w)=p(y∣do(x),z,w) if Y⊥Z∣X,W in $G_{\underline{X}\bar{Z}}$, the causal model modified to remove connections entering *X* and leaving *Z*. When applied to our model, it yields:

1. p(Y∣do(α))=p(Y∣α) (because Y⊥α in $G_{\alpha }$);
2. p(α∣do(β))=p(α∣β) (because α⊥β in $G_{\beta }$);
3. p(Y∣do(α),do(β))=p(Y∣α,β) (because Y⊥(α,β) in $G_{\alpha ,\beta }$).

Starting with the definition of the information flow from α to *Y* imposing β, we have that $\begin{array}{cc} I(\alpha \to Y|do(\beta )) & =E_{\beta }\left[\int_{\alpha }p\left(\alpha |do\left(\beta \right)\right)\int_{Y}p\left(Y|do\left(\alpha \right),do\left(\beta \right)\right)\right.\\ & \;\left.\times \log\frac{p(Y|do(\alpha ),do(\beta ))}{\int_{\alpha^{\prime }}p\left(\alpha =\alpha^{\prime }|do\left(\beta \right)\right)p\left(Y|do\left(\alpha^{\prime }\right),do\left(\beta \right)\right)}\;dY\;d\alpha \right]\\ & =E_{\beta }\left[\int_{\alpha }p\left(\alpha |\beta \right)\int_{Y}p\left(Y|\alpha ,\beta \right)\log\frac{p(Y|\alpha ,\beta )}{\int_{\alpha^{\prime }}p\left(\alpha =\alpha^{\prime }|\beta \right)p\left(Y|\alpha^{\prime },\beta \right)}\;dY\;d\alpha \right]\\ & =E_{\beta }\left[\int_{\alpha ,Y}p\left(Y,\alpha |\beta \right)\log\frac{p(Y|\alpha ,\beta )}{p(Y|\beta )}\;dY\;d\alpha \right]\\ & =\int_{\beta }\int_{\alpha ,Y}p\left(Y,\alpha ,\beta \right)\log\frac{p(Y|\alpha ,\beta )}{p(Y|\beta )}\;dY\;d\alpha \;d\beta \\ & =\int_{\beta }p\left(\beta \right)\int_{\alpha ,Y}p\left(Y,\alpha |\beta \right)\log\frac{p(Y|\alpha ,\beta )}{p(Y|\beta )}\;dY\;d\alpha \\ & =\int_{\beta }p\left(\beta \right)\int_{\alpha ,Y}p\left(Y,\alpha |\beta \right)\log\frac{p(Y,\alpha |\beta )}{p(Y|\beta )p(\alpha |\beta )}\;dY\;d\alpha \\ & =I(\alpha ;Y|\beta ). \end{array}$

   □

### 3.3. Objective Function and Its Estimation

Given an input vector $X\in R^{d}$, we aim to find a subset *S* of size *k* that maximizes $I(X_{S};Y|X_{\bar{S}})$.

The most challenging aspect of our framework lies in accurately estimating $I(X_{S};Y|X_{\bar{S}})$. According to Shannon’s chain rule [23], $I(X_{S};Y|X_{\bar{S}})$ can be decomposed as:

(4) $$\begin{array}{cc} I(X_{S};Y|X_{\bar{S}})= & H\left(Y|X_{\bar{S}}\right)-H\left(Y|X\right)\\ = & H\left(Y,X_{\bar{S}}\right)+H\left(X\right)-H\left(X_{\bar{S}}\right)-H\left(Y,X\right), \end{array}$$

where *H* denotes entropy or joint entropy.

In this work, instead of relying on variational approximation or using the popular mutual information neural estimator (MINE) [24], which may make the joint training become unstable or even result in negative mutual information values [25], we use the matrix-based Rényi’s α-order entropy functional [14,26] to estimate different entropy terms in Equation (4). This newly proposed estimator can be simply computed (without density estimation or any auxiliary neural network) and is differentiable, which suits deep learning applications well. For brevity, we directly give the definitions.

**Definition** **2** ([26]). *Let κ:χ×χ↦R be a real-valued positive definite kernel that is also infinitely divisible [27]. Given ${\left\{x_{i}\right\}}_{i=1}^{n}\in \chi$, each $x_{i}$ can be a real-valued scalar or vector, and the Gram matrix $K\in R^{n\times n}$ computed as $K_{ij}=\kappa \left(x_{i},x_{j}\right)$, a matrix-based analog to Rényi’s α-entropy, can be given by the following functional:*

(5) $$\begin{array}{cc} H_{\alpha }\left(A\right) & =\frac{1}{1-\alpha }\log_{2}\left(\mathrm{tr}(A^{\alpha })\right)\\ & =\frac{1}{1-\alpha }\log_{2}\left(\sum_{i=1}^{n}\lambda_{i}{\left(A\right)}^{\alpha }\right), \end{array}$$

*where δ∈(0,1)∪(1,∞). A is the normalized K, i.e., A=K/tr(K). $\lambda_{i}\left(A\right)$ denotes the i-th eigenvalue of A.*

**Definition** **3** ([14]). *Given a set of n samples ${\left\{x_{i},y_{i},z_{i}\right\}}_{i=1}^{n}$, each sample contains three measurements, x∈χ, y∈γ, and z∈ϵ, obtained from the same realization. Given positive definite kernels $\kappa_{1}:\chi \times \chi \mapsto R$, $\kappa_{2}:\gamma \times \gamma \mapsto R$, and $\kappa_{3}:\epsilon \times \epsilon \mapsto R$, a matrix-based analog to Rényi’s α-order joint-entropy can be defined as:*

(6) $$H_{\alpha }\left(A,B,C\right)=H_{\alpha }\left(\frac{A\circ B\circ C}{\mathrm{tr}(A\circ B\circ C)}\right),$$

*where $A_{ij}=\kappa_{1}\left(x_{i},x_{j}\right)$, $B_{ij}=\kappa_{2}\left(y_{i},y_{j}\right)$, $C_{ij}=\kappa_{3}\left(z_{i},z_{j}\right)$, and A∘B∘C denotes the Hadamard product between matrices A, B, and C.*

Now, given ${\left\{x_{s,i},y_{i},x_{\bar{s},i}\right\}}_{i=1}^{M}$ in a mini-batch of *M* samples ($x_{s,i}$ and $x_{\bar{s},i}$ refer to, respectively, the selected feature subset and its complement subset for the *i*-th instance), we first need to evaluate three Gram matrices, $K_{s}=\kappa \left(x_{s,i},x_{s,j}\right)\in R^{M\times M}$, $K_{y}=\kappa \left(y_{i},y_{j}\right)\in R^{M\times M}$, and $K_{\bar{s}}=\kappa \left(x_{\bar{s},i},x_{\bar{s},j}\right)\in R^{M\times M}$, associated with variables $X_{S}$, *Y*, and $X_{\bar{S}}$, respectively. Based on Definitions 2 and 3, the entropy and joint entropy terms in Equation (4), all can be simply computed over the eigenspectrum of $K_{s}$, $K_{y}$, $K_{\bar{s}}$, or their Hadamard product. Hence, our final estimator is expressed as:

(7) $$\hat{I}\left(X_{S};Y|X_{\bar{S}}\right)=H_{\alpha }\left(K_{y},K_{\bar{s}}\right)+H_{\alpha }\left(K_{s},K_{\bar{s}}\right)-H_{\alpha }\left(K_{\bar{s}}\right)-H_{\alpha }\left(K_{s},K_{y},K_{\bar{s}}\right).$$

Throughout this work, we choose α=1.01 [14,26] and use the radial basis function (RBF) kernel $\kappa \left(x_{i},x_{j}\right)=\exp\left(-\frac{∥x_{i}-x_{j}{∥}^{2}}{2\sigma^{2}}\right)$ to obtain the Gram matrices. For each sample, we evaluate its *k*-nearest distances and take the mean. We choose kernel width σ as the average of the mean values for all samples.

As can be seen, this new family of estimators avoids explicit estimation of the underlying data distributions, making it particularly attractive for challenging problems involving high-dimensional data. In practice, computing the gradient of $I_{\alpha }\left(A;C|B\right)$ is straightforward using any automatic differentiation framework, such as PyTorch (version 1.13.1) [28]. We recommend PyTorch, as its computed gradients are consistent with the analytical ones.

### 3.4. Continuous Subset Sampling

Exhaustively searching over all possible feature subsets of size *k* involves evaluating dk combinations, which is computationally intractable for even moderately sized *d*. To address this challenge, we employ a continuous relaxation strategy based on the Gumbel–Softmax distribution, allowing us to approximate discrete subset sampling in a differentiable manner. Specifically, we independently sample *k* features from the full set of *d* candidates using a stochastic softmax parameterization.

We now describe the architecture of our built-in explainer. Given an input instance $x_{i}\in R^{d}$ and a predefined subset size *k*, we denote by ${℘}_{k}=\{s_{i}\subseteq \left\{1,\ldots ,d\right\}:|s_{i}\left|=k\right\}$ the space of all possible feature subsets of cardinality *k*. Our goal is to identify the subset $s_{i}\in {℘}_{k}$ that most faithfully accounts for the model’s prediction on $x_{i}$. To this end, we define a scoring function $S:R^{d}\to R^{d}$ that assigns an importance weight to each feature. These scores, produced by a neural selector network (as illustrated in Figure 1), reflect the probability of each feature being included in the selected subset. The final subset is then sampled based on these learned importance scores using the Gumbel–Softmax reparameterization.

However, a direct estimation requires summing over dk combinations of feature subsets, which is intractable. Therefore, we employ the Gumbel–Softmax [3,15] to overcome this problem in a differentiable manner. Suppose we aim to approximate a categorical random variable represented as a one-hot vector in $R^{d}$ with category probabilities $p_{1},p_{2},\ldots ,p_{d}$. We start by adding a random perturbation to the log probability of each category $\log p_{i}$:

(8) $$\begin{array}{cc} G_{i}= & -\log\left(-\log u_{i}\right)\;\mathrm{where}\;\;u_{i}∼\mathrm{Uniform}(0,1),\\ \; & C_{i}=\frac{\exp\{\left(G_{i}+\log p_{i}\right)/\tau \}}{\sum_{i=1}^{d}\exp\left\{\left(G_{i}+\log p_{i}\right)/\tau \right\}}, \end{array}$$

where τ is a tuning parameter for the temperature of the Gumbel–Softmax distribution (in our paper, we set τ=0.1). Then, we can define a Concrete random vector $C=[C_{1},\ldots ,C_{d}]$, which serves as a continuous, differential approximation of a categorical random variable represented as a one-hot vector in $R^{d}$.

We further define a continuous-relaxed random variable $C^{*}=\left[C_{1}^{*},\ldots ,C_{d}^{*}\right]$ as the element-wise maximum of the independently sampled Concrete vectors $C^{\left(j\right)}$ where j=1,…,k:

(9) $$C_{i}^{*}=\max_{j}C_{i}^{\left(j\right)}\;\;i.i.d\;\;\mathrm{for}\;\;j=1,\ldots ,k.$$

We denote this sampling result $C^{*}$ as the generated explanation $e_{i}^{m}\in R^{d}$, where the top-*k* features are most informative for the prediction of the model.

## 4. Experiments and Results

### 4.1. Experimental Setup

In this section, we conduct both quantitative and qualitative comparisons between our method and four competing approaches: two state-of-the-art instance-wise feature selection approaches, L2X [6] and INVASE [7], as well as two pixel-level attribution methods, GradCAM [17] and Saliency [16]. Experiments are performed on the MNIST, Fashion-MNIST, and CIFAR datasets.

Both L2X and INVASE are information-theoretic approaches. Specifically, L2X aims to maximize the mutual information objective $I(X_{S};Y)$, while INVASE minimizes the Kullback–Leibler divergence $D_{\mathrm{KL}}\left(p\left(Y|X\right)\;∥\;p\left(Y|X_{S}\right)\right)$. Our objective, based on the conditional mutual information $I(X_{S};Y|X_{\bar{S}})$, is closely related to that of L2X. However, we emphasize that L2X does not establish a connection between its objective and causality. That is, there is no theoretical guarantee that it identifies features that are causally responsible for the model’s prediction.

In contrast to L2X and INVASE, which rely on variational approximations to estimate mutual information, our method introduces a matrix-based Rényi’s entropy functional, enabling a more efficient and principled estimation.

Our method operates at the level of *superpixels* or *image patches*, selecting informative patches instead of individual pixels. To ensure a fair comparison with the pixel-level attribution baselines, we compute the average attribution score within each patch and then select the top-*k* patches accordingly. We use *post-hoc accuracy* [5] for quantitative assessment, which measures predictive consistency using selected features:

$$\mathrm{Accuracy}=\frac{1}{|X_{\mathrm{val}}|}\sum_{x\in X_{\mathrm{val}}}I\left[\arg\max F\left(x\right)=\arg\max F\left(x_{S}\right)\right].$$

### 4.2. Quantitative Results

For all datasets, we report the mean and standard deviation of post-hoc accuracy over five independent runs for all competing approaches. In the following tables, *k* denotes the number of selected 4×4 image patches. GradCAM and Saliency typically do not report variance in this context because they are deterministic methods: given a fixed trained model and a fixed input, they always produce the same output.

#### 4.2.1. MNIST

The MNIST dataset consists of 28×28 grayscale images of handwritten digits. We consider a binary classification task using digits “3” and “8”. In addition, we design four more binary tasks to explore diverse visual distinctions: “1” vs. “7” (vertical vs. angular strokes), “0” vs. “6” (loops with or without tails), “2” vs. “5” (confusingly similar curves), and “4” vs. “9” (sharp angles vs. loops).

A simple convolutional neural network (CNN), composed of two convolutional layers followed by one fully connected layer, is trained as the black-box model and achieves a test accuracy of over 99%. The selector network in L2X, INVASE, and our method is implemented as a three-layer fully convolutional network. For L2X, the variational approximator is parameterized using the same architecture as the black-box model across all experiments.

In detail, the selector network consists of three convolutional blocks designed to process single-channel 28×28 input images. The first block applies a 3×3 convolution with 5 output filters and padding of 2, followed by 2×2 max pooling and a ReLU activation. The second block performs another 3×3 convolution with 16 filters and padding of 2, again followed by 2×2 max pooling and ReLU activation. The final block is a 1×1 convolution that projects the 16-channel feature map to a single channel without any additional non-linearity. The resulting tensor is reshaped to $\left(\mathrm{batch}\;\mathrm{size},k^{2}\right)$, corresponding to a k×k grid of logits that can be interpreted as spatial selection scores.

**Figure 3:**
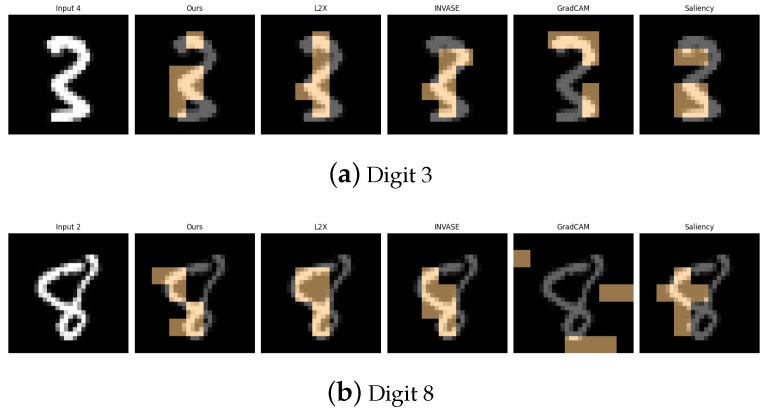
Explanation visualizations of all competing methods on query images for the task of classifying digits 3 and 8. While both digits contain two stacked curves, the digit “8” forms a fully enclosed shape with connected loops, whereas “3” consists of two open arcs separated by a visible gap. Our method distinguishes between open and closed double-loop structures, offering improved interpretability.

**Figure 4:**
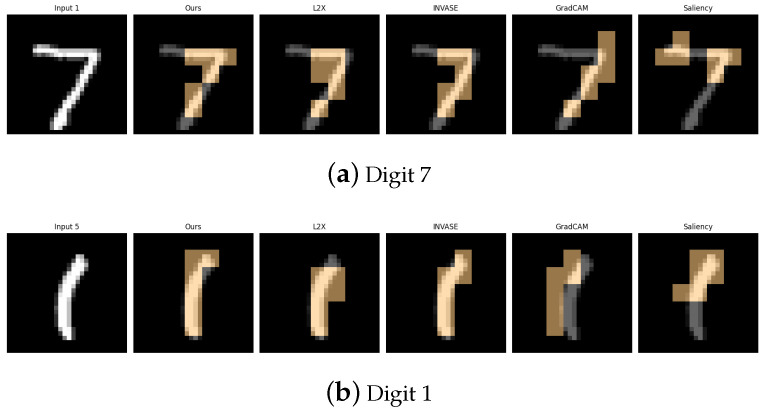
Explanation visualizations of all competing methods on query images for the task of classifying digits 1 and 7. Both digits are simple and share a similar top-down structure, but “1” is primarily a straight vertical line, whereas “7” includes an additional horizontal top stroke. Our method distinguishes between *vertical* and *angular* strokes, offering a more interpretable explanation of the model’s decision-making process.

**Figure 5:**
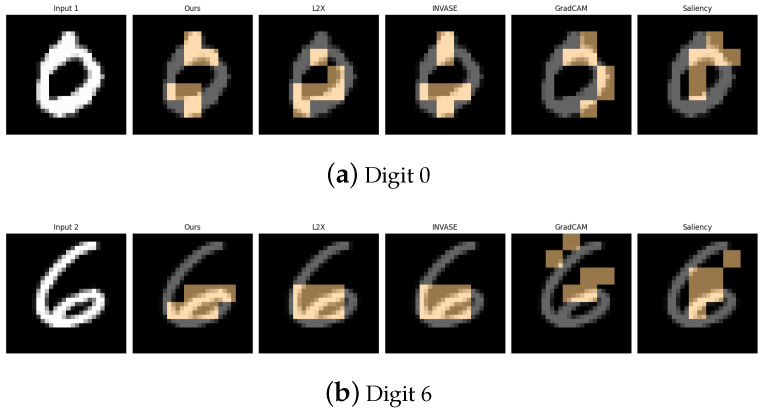
Explanation visualizations of all competing methods on query images for the task of classifying digits 0 and 6; “0” forms a perfect enclosed loop, whereas “6” introduces an additional tail or swirl at the bottom. Our method distinguishes between *pure loops and loop-tail structures*, effectively detecting such nuanced differences and offering more interpretable explanations.

**Figure 6:**
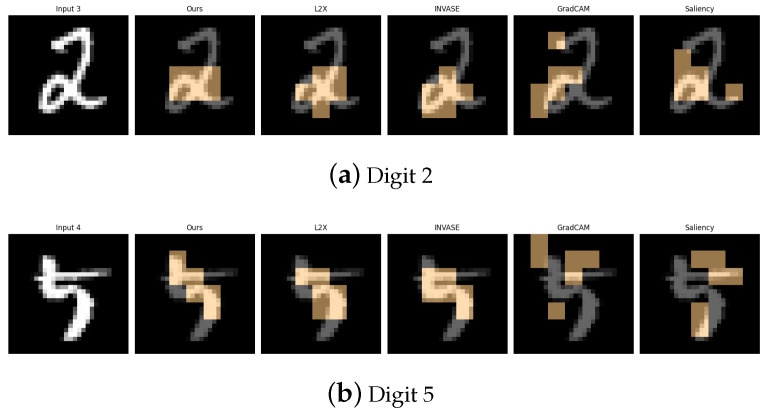
Explanation visualizations of all competing methods on query images for the task of classifying digits 2 and 5; “2” and “5” often appear visually similar in handwritten form, making them a challenging pair. Both digits contain multiple curves and sharp angles, requiring precise analysis of subtle geometric differences. Our method excels at the discrimination of fine-grained structures, leading to more interpretable and accurate explanations.

**Figure 7:**
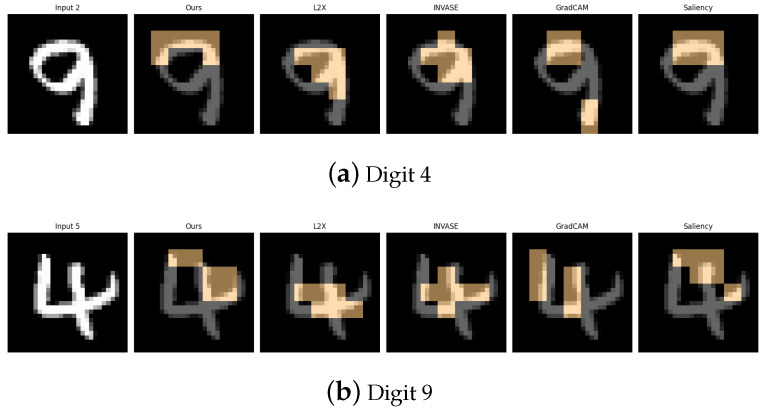
Explanation visualizations of all competing methods on query images for the task of classifying digits 4 and 9; “4” is typically composed of straight lines, whereas “9” features a loop connected to a vertical stem. Our method effectively distinguishes between linear and curved structures, enabling the detection of both straight lines and enclosed loops for more interpretable decision-making.

Table 1, Table 2, Table 3, Table 4 and Table 5 summarize the post-hoc accuracy results, while Figure 3, Figure 4, Figure 5, Figure 6 and Figure 7 visualize the explanations generated by all competing approaches. Our method consistently achieves the highest accuracy across tasks and produces explanations that align closely with human perceptual understanding.

#### 4.2.2. Fashion-MNIST

The Fashion-MNIST dataset [29] consists of 28×28 grayscale images of fashion items such as T-shirts, shoes, and handbags. For our experiments, we focus on a binary classification task involving class 0 (T-shirts) and class 9 (ankle boots). In addition, we design four supplementary binary classification tasks to explore a range of semantic and structural variations: (1) class 0 vs. class 2 (T-shirt vs. pullover), highlighting subtle differences among upper-body garments; (2) class 5 vs. class 9 (sandal vs. ankle boot), contrasting open and closed footwear styles; (3) class 8 vs. class 4 (bag vs. coat), testing the model’s ability to differentiate between accessories and outerwear; and (4) class 1 vs. class 3 (trouser vs. dress), emphasizing the distinction between symmetric and asymmetric lower-body apparel. These carefully chosen pairs allow us to assess model performance across diverse and challenging visual boundaries.

We use the same convolutional neural network architecture as in the MNIST experiments, achieving a test accuracy over 99%. The selector networks in both L2X and our method are implemented using a three-layer fully convolutional architecture.

**Figure 8:**
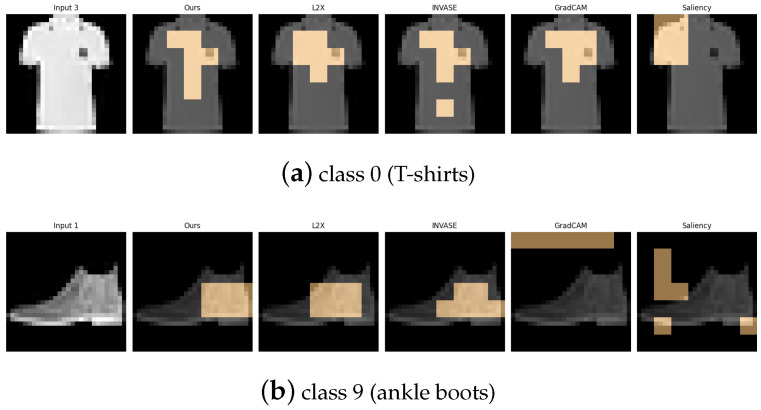
Explanation visualizations of all competing methods on query images for the task of classifying class 0 (T-shirts) and class 9 (ankle boots). Our approach effectively distinguishes between upper-body and lower-body garments by capturing clear structural differences, such as soft fabric versus rigid materials.

**Figure 9:**
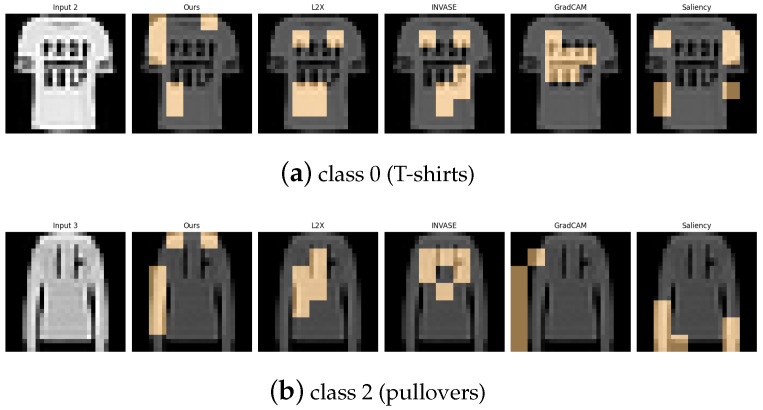
Explanation visualizations of all competing methods on query images for the task of classifying class 0 (T-shirts) and class 2 (pullovers). Class “0” (T-shirt) and class “2” (pullover) represent similar upper-body garments with subtle visual differences: T-shirts typically have shorter sleeves and a looser fit, whereas pullovers usually feature long sleeves and a more uniform texture. Our method effectively distinguishes between these characteristics, particularly sleeve length, resulting in higher accuracy and explanations that closely align with human perception.

**Figure 10:**
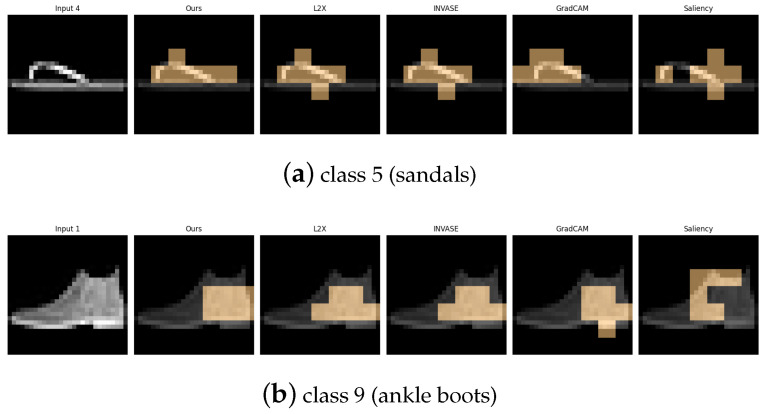
Explanation visualizations of all competing methods on query images for the task of classifying class 5 (sandals) and class 9 (ankle boots). Class “5” (sandal) and class “9” (ankle boot) represent two types of footwear with distinct visual characteristics—sandals are typically open-toed and lightweight, while ankle boots are enclosed and rigid. Our method effectively captures these structural differences, especially the presence or absence of open regions, leading to superior classification accuracy and interpretable attributions.

**Figure 11:**
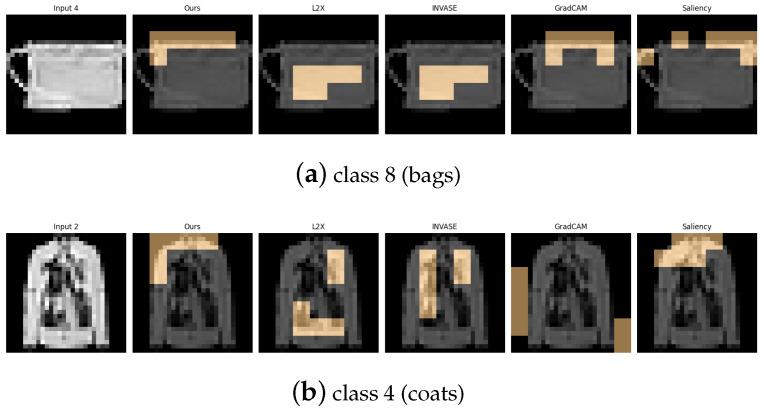
Explanation visualizations of all competing methods on query images for the task of classifying class 8 (bags) and class 4 (coats). Class “8” (bag) and class “4” (coat) represent visually and functionally distinct categories—bags are typically compact and symmetric with handles, while coats are elongated garments with sleeves and complex contour lines. Our method captures these differences effectively, identifying key visual cues such as shoulder width and the presence of handles, resulting in accurate and interpretable predictions.

**Figure 12:**
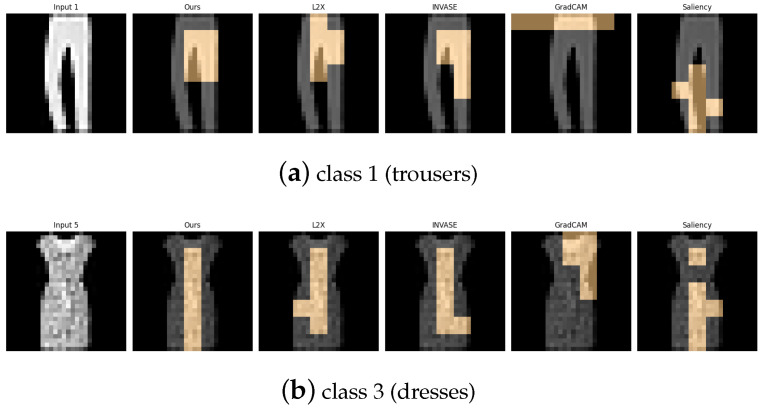
Explanation visualizations of all competing methods on query images for the task of classifying class 1 (trousers) and class 3 (dresses). Our method demonstrates strong structural awareness when distinguishing between class “1” (trousers) and class “3” (dresses). For trousers, the explanation highlights the central hollow region between the two legs and the overall bilateral symmetry, key visual cues that align with human perception. In contrast, for dresses, which typically have a continuous lower contour, no such hollow region is detected. Instead, the explanation focuses on the single-piece flowing silhouette. These results illustrate the interpretability of our method in capturing meaningful structural differences between garment types.

Table 6, Table 7, Table 8, Table 9 and Table 10 summarize the post-hoc accuracy results, while Figure 8, Figure 9, Figure 10, Figure 11 and Figure 12 visualize the explanations generated by all competing approaches. Our method consistently achieves the highest accuracy across tasks and produces explanations that align closely with human perceptual understanding.

#### 4.2.3. CIFAR-10

The CIFAR-10 dataset [30] consists of 60,000 32×32 RGB images spanning 10 object categories. For our experiments, we focus on a binary classification task involving class 2 (birds) and class 9 (trucks), which represent a high-level semantic contrast between natural and man-made objects. Additionally, we design three supplementary binary tasks to capture various types of visual and semantic diversity: (1) class 0 vs. class 1 (airplane vs. automobile), contrasting aerial and ground transportation modes; (2) class 4 vs. class 7 (deer vs. horse), comparing quadrupedal mammals with differing body shapes and textures; and (3) class 6 vs. class 8 (frog vs. ship), contrasting natural amphibians with large-scale artificial structures. These pairings are carefully selected to challenge the model across a spectrum of low-level (e.g., color, shape) and high-level (e.g., semantics, context) visual cues.

We train a three-layer convolutional neural network as the black-box model, achieving 94% test accuracy. The selector networks used in both L2X and our method are implemented as three-layer fully convolutional networks.

**Figure 13:**
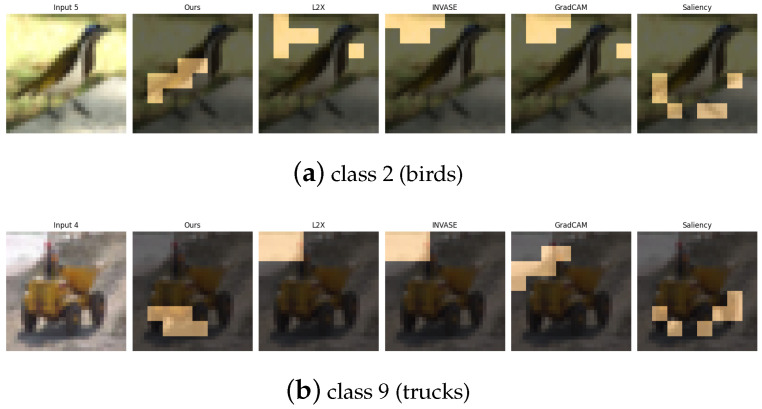
Explanation visualizations of all competing methods on query images for the task of classifying class 2 (birds) and class 9 (trucks). Class “2” (birds) and class “9” (trucks) represent a high-level semantic contrast between natural and man-made objects. Birds are characterized by organic shapes, wings, and smooth contours, while trucks exhibit rigid structures with prominent components such as wheels and chassis. Our method accurately captures these key features by highlighting the wings and body contours of birds and the wheels and lower structure of trucks, resulting in both high classification accuracy and human-aligned interpretability.

**Figure 14:**
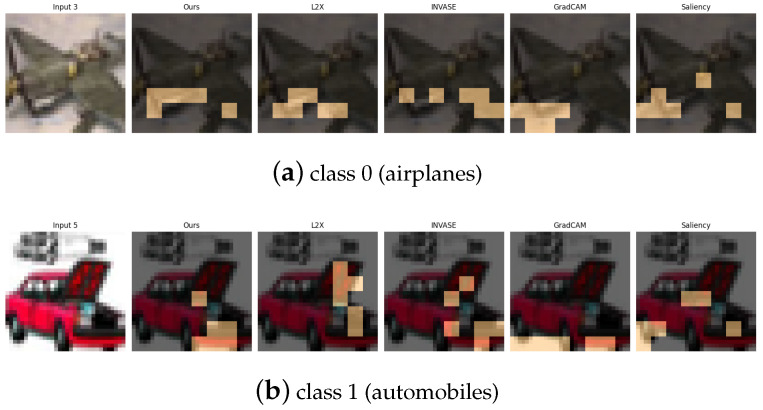
Explanation visualizations of all competing methods on query images for the task of classifying class 0 (airplanes) and class 1 (automobiles). Class “0” (airplane) and class “1” (automobile) represent distinct categories of vehicles operating in different environments—air and ground. Airplanes are characterized by elongated fuselages, wings, and, in some cases, visible propellers, while automobiles are defined by compact bodies and wheels. Our method effectively captures these discriminative features, such as airplane wings and propellers and automobile wheels and chassis, resulting in high classification accuracy and semantically meaningful, human-aligned explanations.

**Figure 15:**
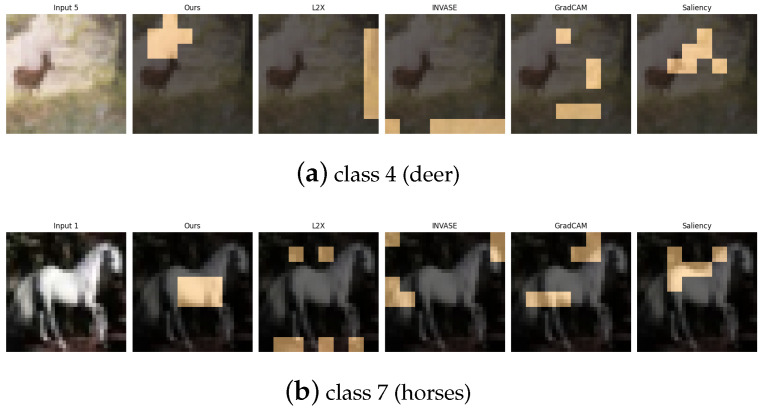
Explanation visualizations of all competing methods on query images for the task of classifying class 4 (deer) and class 7 (horses). Class “4” (deer) and class “7” (horse) both represent four-legged mammals with similar body plans, but differ in several fine-grained structural features. Our method demonstrates strong interpretability by highlighting class-specific anatomical and textural cues. For deer, it attends to the antlers, a distinctive feature not present in horses. For horses, it focuses on the solid-colored underbelly region. These explanations not only align well with human visual intuition but also reveal biologically meaningful cues that contribute to accurate and interpretable predictions.

**Figure 16:**
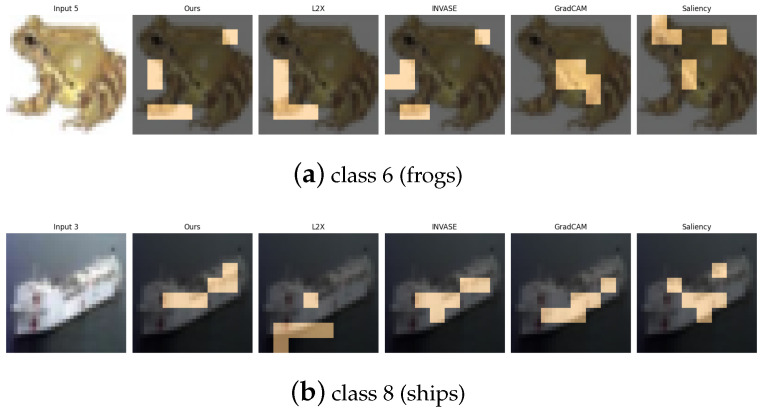
Explanation visualizations of all competing methods on query images for the task of classifying class 6 (frogs) and class 8 (ships). Class “6” (frog) and class “8” (ship) represent a contrast between natural and artificial objects. Frogs typically exhibit irregular, organic shapes, soft textures, and limb contours, while ships are rigid, geometrically structured, and often appear with horizontal decks. Our method captures these key differences effectively by focusing on features such as the leg outlines of frogs and the deck structures of ships, leading to improved performance and interpretable, semantically coherent explanations.

Table 11, Table 12, Table 13 and Table 14 summarize the post-hoc accuracy results, while Figure 13, Figure 14, Figure 15 and Figure 16 visualize the explanations generated by all competing approaches. Our method consistently achieves the highest accuracy across tasks and produces explanations that align closely with human perceptual understanding.

### 4.3. Ablation Study

We finally provide an ablation study to evaluate the sensitivity of our method with respect to the entropy order α used in the matrix-based Rényi’s α-order entropy functional. Specifically, we varied α from 0.5 to 5 in increments of 0.5 to perform binary classification between digits 3 and 8 in the MNIST dataset. The results, summarized in Table 15, show that our method consistently outperforms INVASE (the second-best method) across a wide range of α values. This indicates that our framework is robust to the choice of α, and maintains strong performance even as the entropy order varies, especially in the range α∈(0.5,2).

## 5. Conclusions and Future Work

We introduce a new approach for interpreting deep vision models via causally grounded instance-wise feature selection. By aligning feature subsets with maximal conditional influence on outputs, our method produces compact and informative explanations. Experimental results demonstrate superior performance in causal effect and predictive alignment, showing promise for real-world deployment.

Future work is twofold. On the one hand, although this paper employs the matrix-based Rényi’s α-order entropy functional to effectively estimate the conditional mutual information term without requiring density estimation or variational approximation, the computational complexity remains $O(M^{3})$, where *M* is the mini-batch size. In future work, we plan to adopt a fast computation scheme for the matrix-based Rényi’s α-order entropy functional [31], which leverages randomized numerical linear algebra techniques and reduces the complexity to below $O(M^{2})$.

On the other hand, while our method is primarily demonstrated on visual classification tasks, its core framework maximizes a differentiable estimate of the conditional mutual information (CMI) between selected features and model outputs, is modality-agnostic, and can be conceptually extended to other domains such as text or tabular data. The matrix-based Rényi entropy estimator used for CMI depends only on positive-definite kernels and does not rely on domain-specific assumptions, which makes it applicable beyond image data.

For text data, individual tokens can be treated as features [3], with masking performed by replacing tokens with a special [MASK] token or by dropping their embeddings. For tabular data, features correspond to columns or column groups, and masking can be implemented by substituting values with their mean or a designated sentinel value. In both cases, the explainer network and kernel function can be adapted to the data modality, while the overall training and optimization pipeline remains unchanged.

## Figures and Tables

**Table 1:** Post-hoc accuracy (MNIST, 3 and 8). ** indicates statistically significant improvement over L2X at p<0.01. The best performance is in bold.

Method	*k* = 4	*k* = 6	*k* = 8
Ours	0.951±0.007 **	0.974±0.004 **	0.984±0.004 **
INVASE	0.945±0.006	0.969±0.005	0.982±0.004
L2X	0.940±0.007	0.967±0.005	0.979±0.003
GradCAM	0.802	0.829	0.842
Saliency	0.866	0.921	0.956

**Table 2:** Post-hoc accuracy (MNIST, 1 and 7). ** indicates statistically significant improvement over L2X at p<0.01. The best performance is in bold.

Method	*k* = 4	*k* = 6	*k* = 8
Ours	0.943±0.006 **	0.967±0.004 **	0.978±0.005 **
INVASE	0.936±0.005	0.961±0.006	0.975±0.004
L2X	0.929±0.007	0.958±0.005	0.972±0.003
GradCAM	0.794	0.821	0.838
Saliency	0.859	0.910	0.949

**Table 3:** Post-hoc accuracy (MNIST, 0 and 6). ** indicates statistically significant improvement over L2X at p<0.01. The best performance is in bold.

Method	*k* = 4	*k* = 6	*k* = 8
Ours	0.949±0.006 **	0.971±0.004 **	0.982±0.005 **
INVASE	0.941±0.007	0.965±0.005	0.978±0.004
L2X	0.934±0.006	0.961±0.005	0.974±0.003
GradCAM	0.790	0.818	0.835
Saliency	0.855	0.908	0.946

**Table 4:** Post-hoc accuracy (MNIST, 2 and 5). ** indicates statistically significant improvement over L2X at p<0.01. The best performance is in bold.

Method	*k* = 4	*k* = 6	*k* = 8
Ours	0.946±0.007 **	0.968±0.004 **	0.980±0.004 **
INVASE	0.938±0.006	0.962±0.005	0.976±0.004
L2X	0.931±0.007	0.958±0.005	0.973±0.003
GradCAM	0.788	0.815	0.831
Saliency	0.850	0.903	0.943

**Table 5:** Post-hoc accuracy (MNIST, 4 and 9). ** indicates statistically significant improvement over L2X at p<0.01. The best performance is in bold.

Method	*k* = 4	*k* = 6	*k* = 8
Ours	0.944±0.006 **	0.969±0.004 **	0.981±0.004 **
INVASE	0.936±0.007	0.964±0.005	0.977±0.004
L2X	0.928±0.006	0.959±0.005	0.973±0.003
GradCAM	0.786	0.812	0.830
Saliency	0.848	0.901	0.942

**Table 6:** Post-hoc accuracy (Fashion-MNIST, class 0 (T-shirts) and class 9 (ankle boots)). *, **, and *** indicate statistically significant improvement over L2X at p<0.05, p<0.01, and p<0.001, respectively. The best performance is in bold.

Method	*k* = 4	*k* = 6	*k* = 8
Ours	0.914±0.021 ***	0.967±0.010 *	0.981±0.004 ***
INVASE	0.902±0.019 **	0.962±0.011	0.976±0.009 *
L2X	0.887±0.025	0.960±0.007	0.970±0.012
GradCAM	0.593	0.639	0.681
Saliency	0.561	0.837	0.930

**Table 7:** Post-hoc accuracy (Fashion-MNIST, class 0 (T-shirts) and class 2 (pullovers)). *, **, and *** indicate statistically significant improvement over L2X at p<0.05, p<0.01, and p<0.001, respectively. The best performance is in bold.

Method	*k* = 4	*k* = 6	*k* = 8
Ours	0.907±0.020 ***	0.958±0.012 **	0.976±0.006 ***
INVASE	0.895±0.019 **	0.951±0.013	0.971±0.010 *
L2X	0.883±0.024	0.948±0.010	0.967±0.012
GradCAM	0.579	0.631	0.678
Saliency	0.549	0.829	0.921

**Table 8:** Post-hoc accuracy (Fashion-MNIST, class 5 (sandals) and class 9 (ankle boots)). *, **, and *** indicate statistically significant improvement over L2X at p<0.05, p<0.01, and p<0.001, respectively. The best performance is in bold.

Method	*k* = 4	*k* = 6	*k* = 8
Ours	0.919±0.019 ***	0.964±0.011 **	0.982±0.005 ***
INVASE	0.905±0.018 **	0.957±0.012	0.977±0.009 *
L2X	0.893±0.023	0.953±0.011	0.972±0.012
GradCAM	0.601	0.648	0.692
Saliency	0.576	0.843	0.932

**Table 9:** Post-hoc accuracy (Fashion-MNIST, class 8 (bags) and class 4 (coats)). *, **, and *** indicate statistically significant improvement over L2X at p<0.05, p<0.01, and p<0.001, respectively. The best performance is in bold.

Method	*k* = 4	*k* = 6	*k* = 8
Ours	0.926±0.017 ***	0.968±0.009 **	0.984±0.004 ***
INVASE	0.912±0.018 **	0.962±0.010	0.978±0.008 *
L2X	0.898±0.022	0.957±0.009	0.974±0.011
GradCAM	0.606	0.654	0.699
Saliency	0.583	0.849	0.936

**Table 10:** Post-hoc accuracy (Fashion-MNIST, class 1 (trousers) and class 3 (dresses)). *, **, and *** indicate statistically significant improvement over L2X at p<0.05, p<0.01, and p<0.001, respectively. The best performance is in bold.

Method	*k* = 4	*k* = 6	*k* = 8
Ours	0.931±0.015 ***	0.971±0.008 **	0.985±0.004 ***
INVASE	0.918±0.017 **	0.964±0.010	0.980±0.007 *
L2X	0.904±0.021	0.958±0.009	0.975±0.010
GradCAM	0.612	0.662	0.703
Saliency	0.586	0.855	0.938

**Table 11:** Post-hoc accuracy (CIFAR-10, class 2 (birds) and class 9 (trucks)). *, **, and *** indicate statistically significant improvement over L2X at p<0.05, p<0.01, and p<0.001, respectively. The best performance is in bold.

Method	20% Pixels	30% Pixels	40% Pixels
Ours	0.608±0.055 **	0.728±0.047 ***	0.785±0.029 ***
INVASE	0.582±0.042 *	0.693±0.032 **	0.752±0.028 **
L2X	0.513±0.125	0.605±0.012	0.661±0.011
GradCAM	0.576	0.658	0.703
Saliency	0.549	0.574	0.615

**Table 12:** Post-hoc accuracy (CIFAR-10, class 0 (airplanes) and class 1 (automobiles)). *, **, and *** indicate statistically significant improvement over L2X at p<0.05, p<0.01, and p<0.001, respectively. The best performance is in bold.

Method	20% Pixels	30% Pixels	40% Pixels
Ours	0.641±0.048 **	0.755±0.036 ***	0.801±0.027 ***
INVASE	0.624±0.041 *	0.727±0.034 **	0.778±0.028 **
L2X	0.576±0.096	0.684±0.015	0.730±0.014
GradCAM	0.591	0.662	0.703
Saliency	0.563	0.601	0.648

**Table 13:** Post-hoc accuracy (CIFAR-10, class 4 (deer) and class 7 (horses)). *, **, and *** indicate statistically significant improvement over L2X at p<0.05, p<0.01, and p<0.001, respectively. The best performance is in bold.

Method	20% Pixels	30% Pixels	40% Pixels
Ours	0.615±0.046 **	0.736±0.033 ***	0.789±0.027 ***
INVASE	0.598±0.041 *	0.711±0.032 **	0.763±0.028 **
L2X	0.554±0.078	0.662±0.017	0.716±0.015
GradCAM	0.567	0.641	0.687
Saliency	0.547	0.596	0.642

**Table 14:** Post-hoc accuracy (CIFAR-10, class 6 (frogs) and class 8 (ships)). *, **, and *** indicate statistically significant improvement over L2X at p<0.05, p<0.01, and p<0.001, respectively. The best performance is in bold.

Method	20% Pixels	30% Pixels	40% Pixels
Ours	0.633±0.051 **	0.752±0.039 ***	0.801±0.027 ***
INVASE	0.615±0.047 *	0.723±0.036 **	0.774±0.028 **
L2X	0.562±0.091	0.672±0.019	0.719±0.017
GradCAM	0.579	0.644	0.693
Saliency	0.552	0.593	0.641

**Table 15:** Post-hoc accuracy (MNIST 3 vs. 8) for different values of α in our method.

Method	*k* = 4	*k* = 6	*k* = 8
Ours (α=0.5)	0.947 ± 0.006	0.971 ± 0.006	0.981 ± 0.006
Ours (α=1.01)	0.951 ± 0.007	0.974 ± 0.004	0.984 ± 0.004
Ours (α=1.5)	0.948 ± 0.006	0.972 ± 0.006	0.982 ± 0.006
Ours (α=2.0)	0.945 ± 0.006	0.970 ± 0.006	0.978 ± 0.006
Ours (α=2.5)	0.942 ± 0.007	0.968 ± 0.007	0.975 ± 0.007
Ours (α=3.0)	0.939 ± 0.007	0.962 ± 0.007	0.972 ± 0.007
Ours (α=3.5)	0.936 ± 0.008	0.959 ± 0.008	0.969 ± 0.008
Ours (α=4.0)	0.934 ± 0.009	0.956 ± 0.009	0.967 ± 0.009
Ours (α=4.5)	0.931 ± 0.010	0.953 ± 0.010	0.964 ± 0.010
Ours (α=5.0)	0.929 ± 0.011	0.950 ± 0.011	0.962 ± 0.011
INVASE	0.945 ± 0.006	0.969 ± 0.005	0.982 ± 0.004

## Data Availability

Data are contained within the article.
